# Supplementary material for: Investigating the Biological Potency of Nitazoxanide-Based Cu(II), Ni(II) and Zn(II) Complexes Synthesis, Characterization and Anti-COVID-19, Antioxidant, Antibacterial and Anticancer Activities
Source: Molecules. 2023 Aug 18;28(16):6126. doi: 10.3390/molecules28166126 (PMC10458470; doi:10.3390/molecules28166126)
Supplement: Supplementary file 1 [file molecules-28-06126-s001.zip › molecules-2514244-supplementary.pdf]

Investigating the Biological Potency of Nitazoxanide-Based Cu(II), Ni(II) and Zn(II) Complexes Synthesis, Characterization and Anti-COVID-19, Antioxidant, Antibacterial and Anticancer Activities

Abeer A. Sharfalddin, Inas M. Al-Younis, Abdul-Hamid Emwas and Mariusz Jaremko

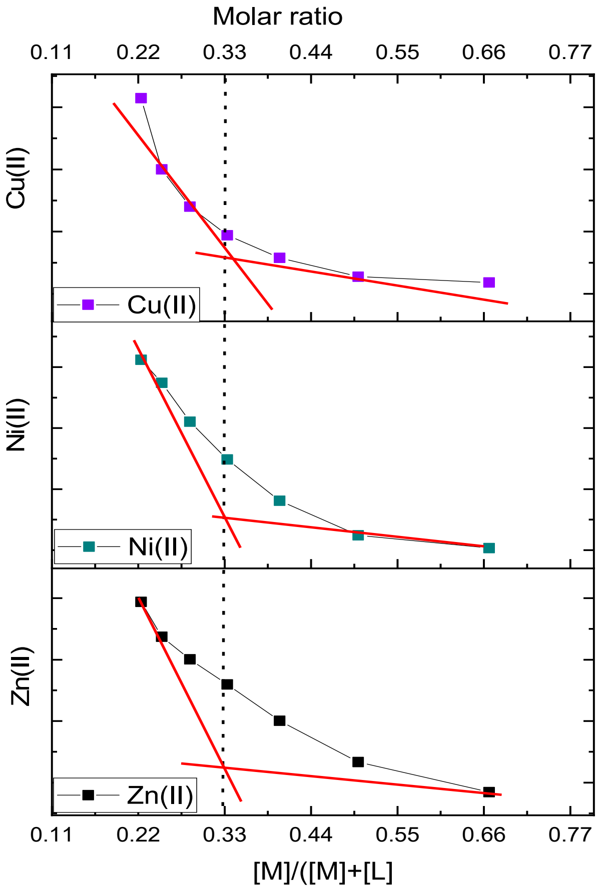

Figure S1. The molar ratio of the obtained metal complexes by plotting the absorbance values toward the ratio of [M]/([M]+[L])

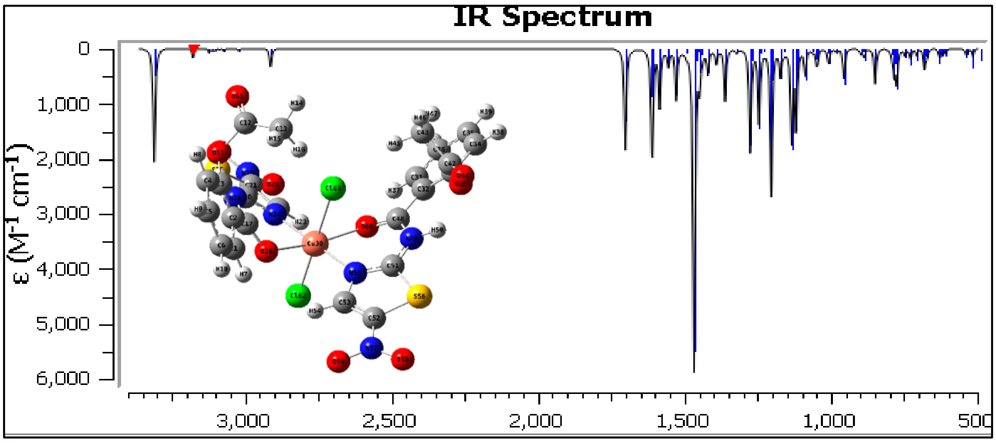
IR Spectrum
ε (M-1 cm-1)
0
1,000
2,000
3,000
4,000
5,000
6,000
3,000
2,500
2,000
1,500
1,000
500

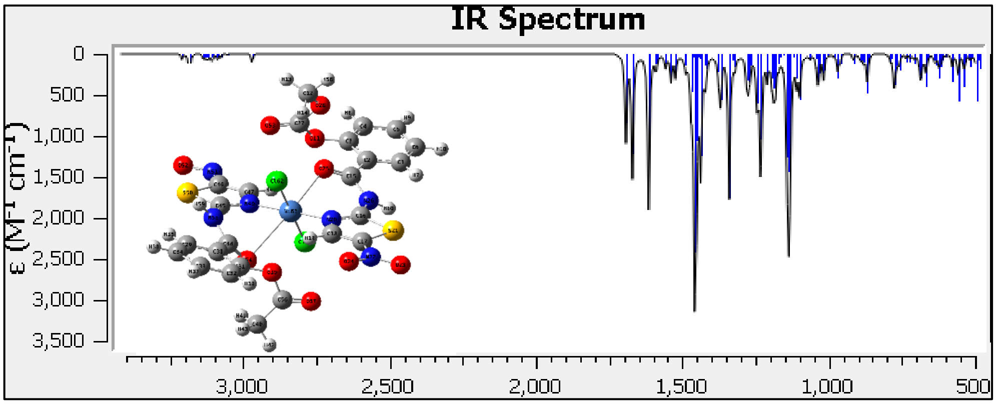
IR Spectrum
ε (M-1 cm-1)
0
500
1,000
1,500
2,000
2,500
3,000
3,500
3,000
2,500
2,000
1,500
1,000
500

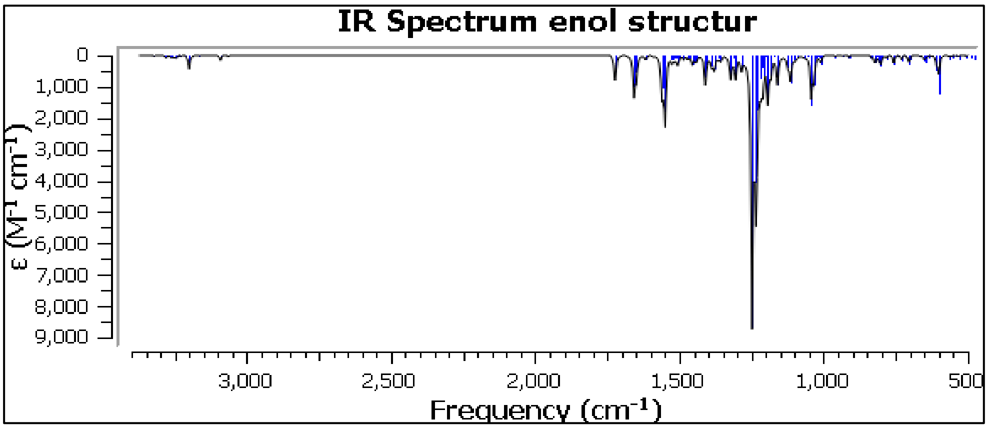
IR Spectrum enol structur
ε (M-1 cm-1)
0
1,000
2,000
3,000
4,000
5,000
6,000
7,000
8,000
9,000
3,000
2,500
2,000
1,500
1,000
500
Frequency (cm-1)

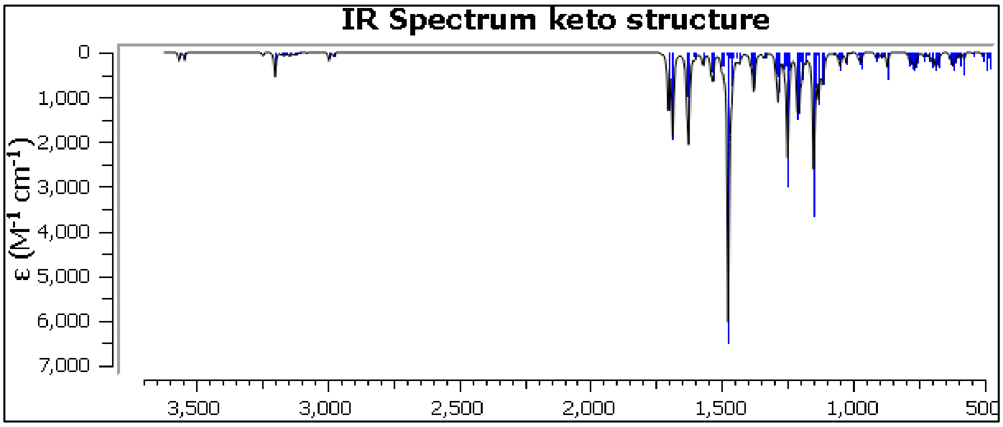
IR Spectrum keto structure
ε (M-1 cm-1)
0
1,000
2,000
3,000
4,000
5,000
6,000
7,000
3,500
3,000
2,500
2,000
1,500
1,000
500

Figure S2. The computed IR spectra of the obtained complexes with enol and keto form of Zn(II) compound and the optimized structure for $Cu(NTZ)_2Cl_2$ and $Ni(NTZ)_2Cl_2$

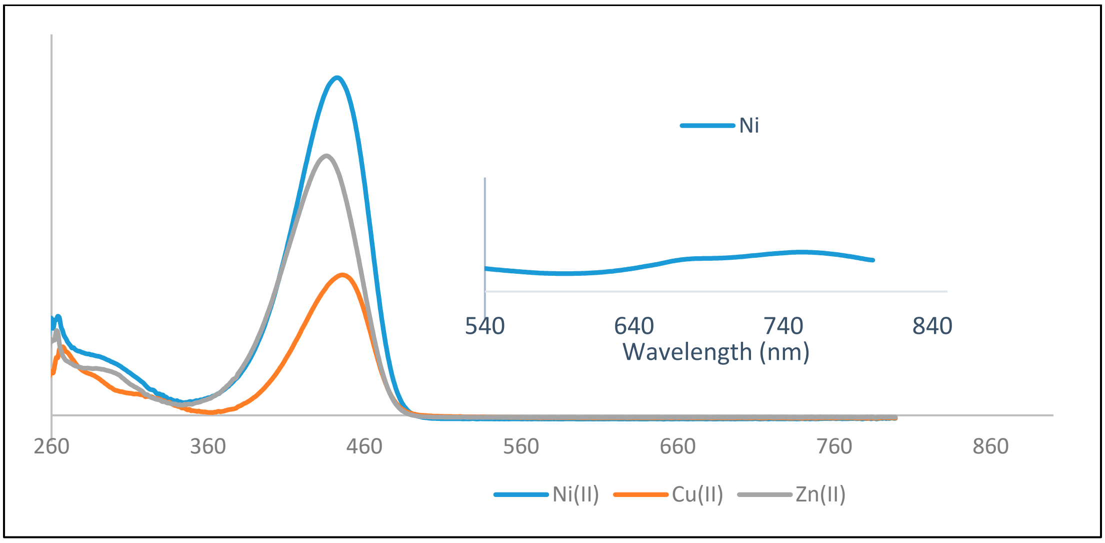

Figure S3. Electronic spectra for the metal complexes.

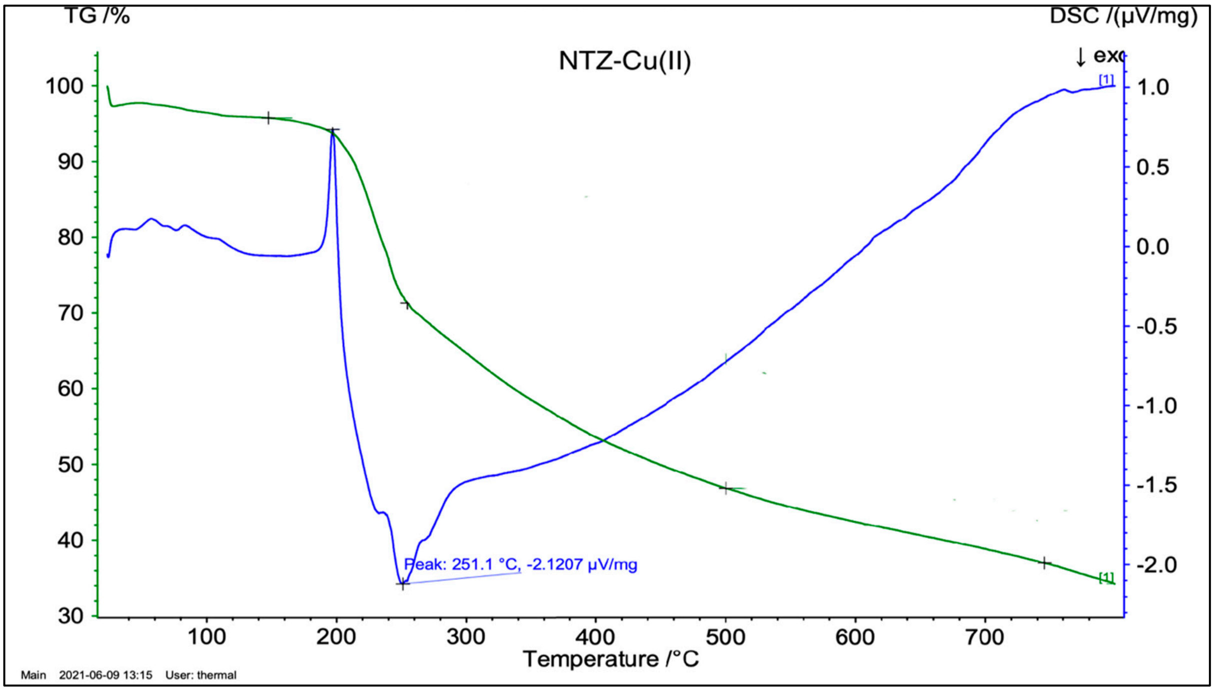

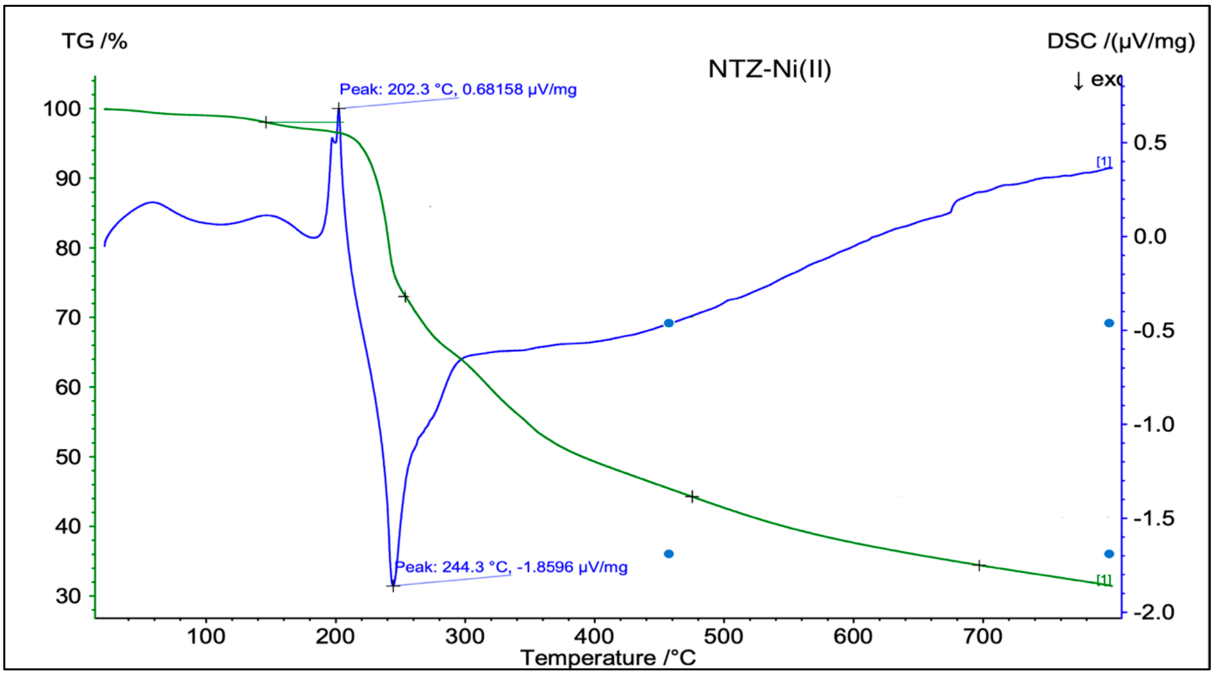

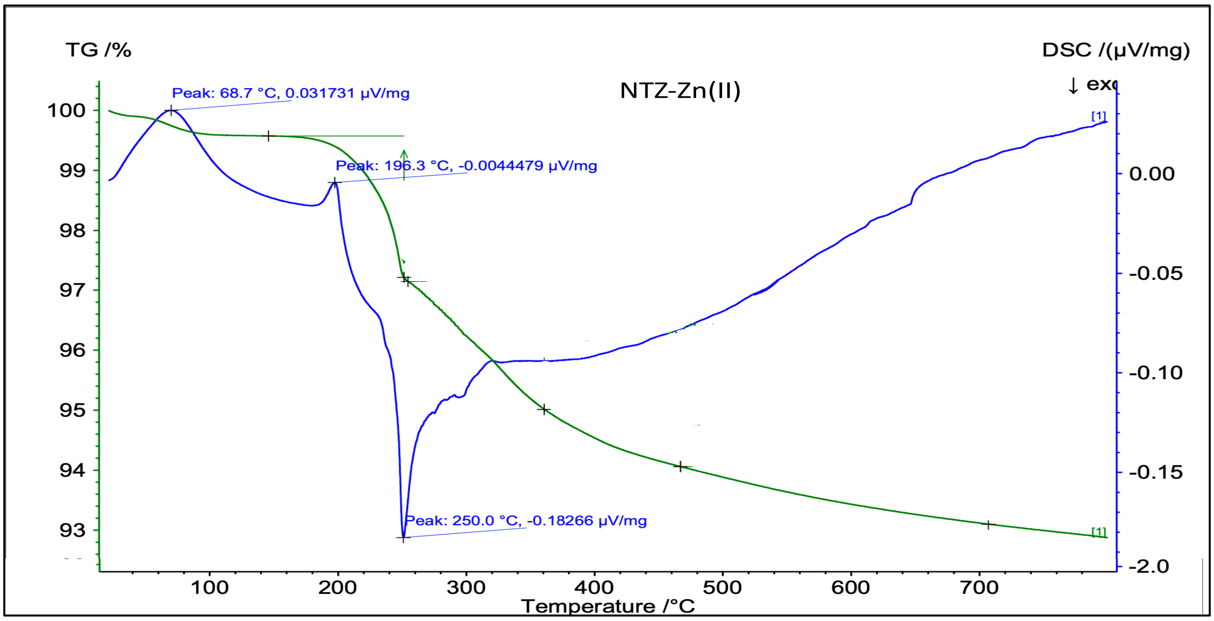

Figure S4. TGA/DSC curves of the synthesized Cu(II), Ni(II) and Zn(II) complexes.

Table S1. Selected geometric bond lengths and bond angles around the metal ion of the optimized complexes

Compound	$[Cu(NTZ)_2Cl_2]$	$[Ni(NTZ)_2Cl_2]$	$[Zn(NTZ)_2Cl_2]$
Bond length			
M-O29	2.35	2.52	2.01
M-N54	2.42	1.90	2.09
M-N24	2.03	1.90	2.63

M-O59	2.03	2.52	-
M-Cl	2.41	2.36	-
Bond angle			
O29-M-N24	80.29	82.15	79.60
N24-M-N54	178.2	178.6	162.27
Cl-M-N24	88.18	90.70	94.76
O29-M-Cl	92.57	88.45	91.36
O25-M-O54	179.86	166.87	160.24
O59-M-N54	78.48	82.15	78.71
O59-M-N24	99.83	97.89	83.57
O59-M-Cl	87.50	88.46	86.10

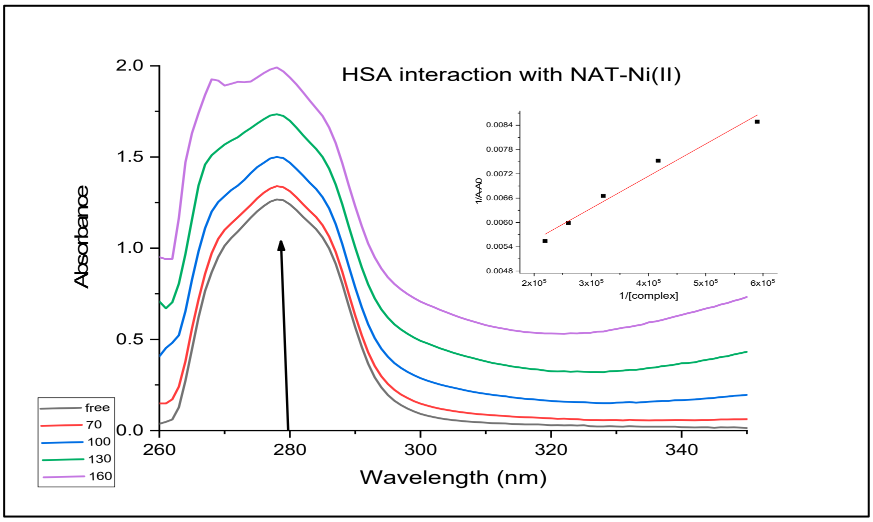

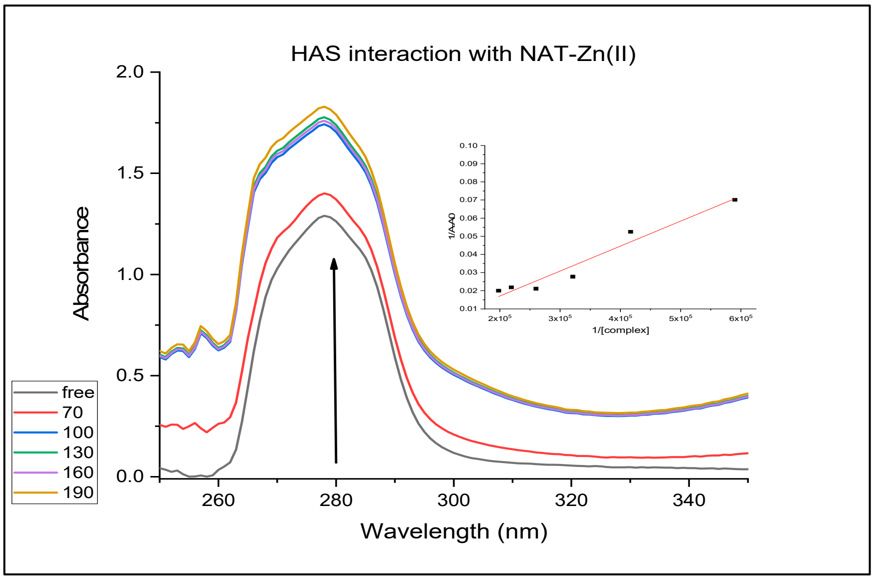

Figure S5. UV–visible absorption spectra of HSA following the titration of increasing the amount of Zn(II) and Ni(II) complexes (70–190 µL) in Tris–HCl buffer solution. Inset: Linear plot of [DNA]/(εa − εf) vs [DNA.

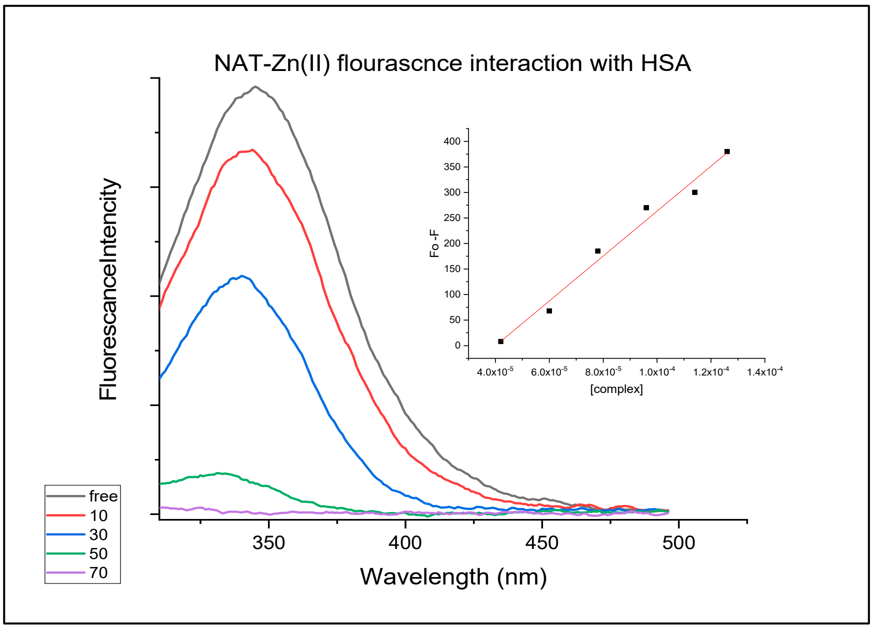

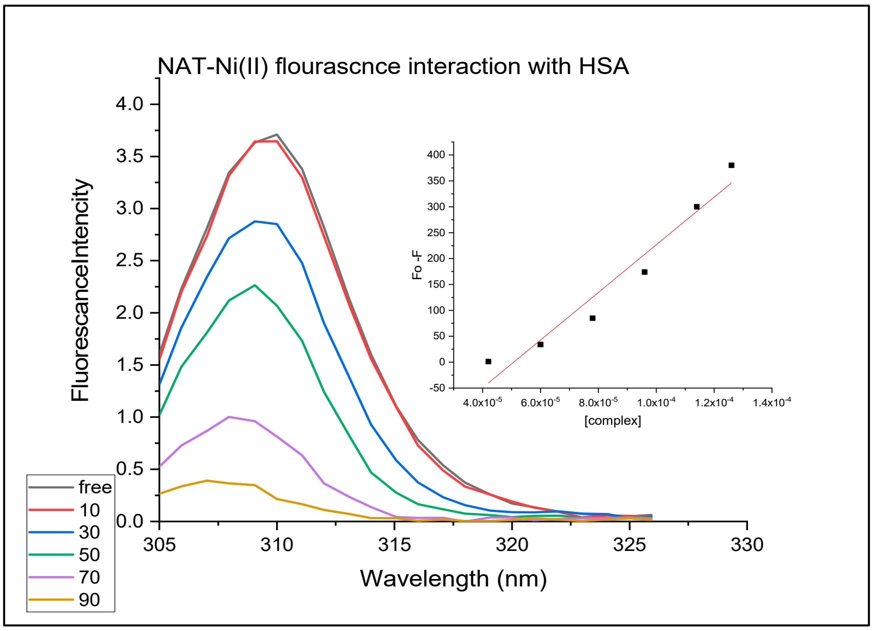

Figure S6. Fluorescence quenching spectra of HSA with rising quantity Zn(II) and Ni(II) complexes (10–90 µM) Inset: Stern–Volmer plot for the quenching intensity of HSA by the metal complexes

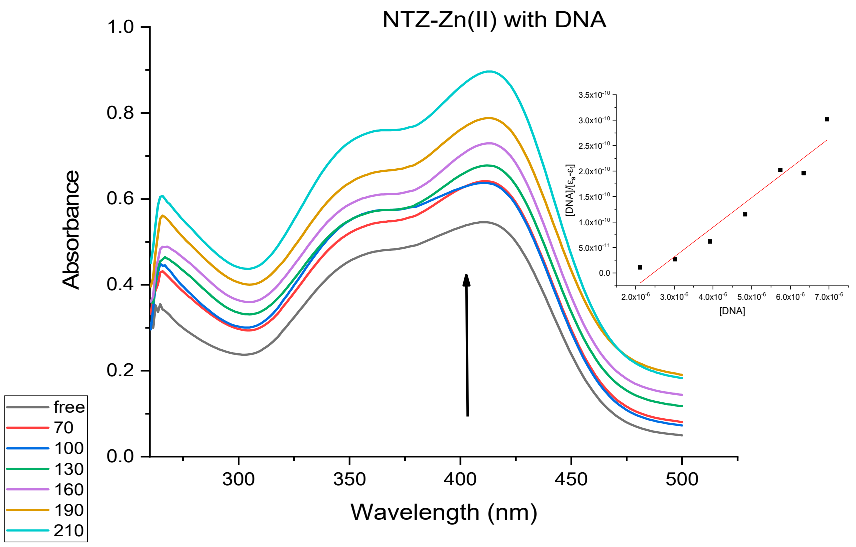

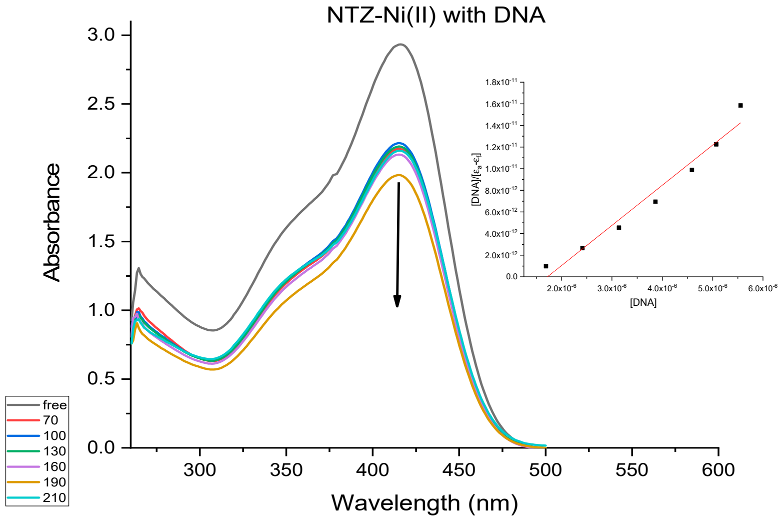

Table S2. Binding score (kcal mol^{-1}) for NTZ and its metal complexes toward SARS-CoV-2 (6LU7) protein receptors.

	Binding affinity for SARS-CoV-2 (6LU7)	RMSD
Ligand	-6.39776	0.893896
Zn	-5.62794	3.299199
Cu	-8.14215	1.738275
Ni	-8.73124	2.195087

Figure S7. The UV–visible titration of NTZ-Ni(II) and NTZ-Zn(II) with increasing the amount of CT-DNA in Tris–HCl buffer solution . Inset: Linear plot of [DNA]/(εa − εf) vs [DNA].

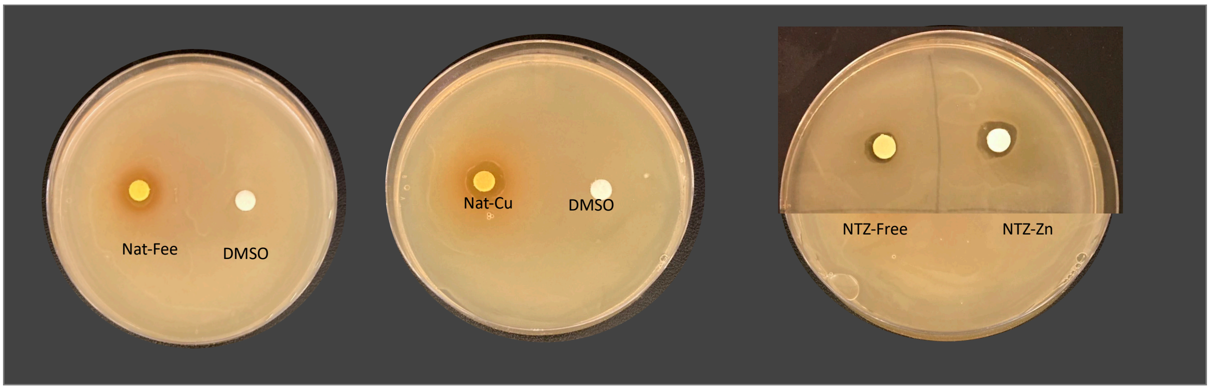

Figure S8. Inhibition zone in agar plate for ligand and its complexes. DMSO used a s a negative control.
